# Multi-Omics Reveal the Efficient Phosphate-Solubilizing Mechanism of Bacteria on Rocky Soil

**DOI:** 10.3389/fmicb.2021.761972

**Published:** 2021-12-09

**Authors:** Yanqiang Ding, Zhuolin Yi, Yang Fang, Sulan He, Yuming Li, Kaize He, Hai Zhao, Yanling Jin

**Affiliations:** ^1^CAS Key Laboratory of Environmental and Applied Microbiology, Environmental Microbiology Key Laboratory of Sichuan Province, Chengdu Institute of Biology, Chinese Academy of Sciences, Chengdu, China; ^2^Sweetpotato Institute, Nanchong Academy of Agricultural Sciences, Nanchong, China

**Keywords:** barren rocky soil, application potential, multi-omics, phosphate-solubilizing bacteria, phosphate-solubilizing mechanism

## Abstract

Phosphate-solubilizing bacteria (PSB) can alleviate available phosphorus (AP)-deficiency without causing environmental pollution like chemical phosphate fertilizers. However, the research and application of PSB on the barren rocky soil is very rare. We screened six PSB from sweetpotato rhizosphere rocky soil. Among them, *Ochrobactrum haematophilum* FP12 showed the highest P-solubilizing ability of 1,085.00 mg/L at 7 days, which was higher than that of the most reported PSB. The assembled genome of PSB FP12 was 4.92 Mb with P-solubilizing and plant growth-promoting genes. In an AP-deficient environment, according to transcriptome and metabolomics analysis, PSB FP12 upregulated genes involved in gluconic acid synthesis and the tricarboxylic acid cycle, and increased the concentration of gluconic acid and malic acid, which would result in the enhanced P-solubilizing ability. Moreover, a series of experiments in the laboratory and field confirmed the efficient role of the screened PSB on significantly increasing AP in the barren rocky soil and promoting sweetpotato yield. So, in this study, we screened highly efficient PSB, especially suitable for the barren rocky soil, and explored the P-solubilizing mechanism. The research will reduce the demand for chemical phosphate fertilizers and promote the environment-friendly agricultural development.

## Introduction

Available phosphorus (AP) is deficient in 74% of the arable soil of China and in more than 40% of the arable soil globally, because about 99% of the total phosphorus (TP) is dissolved (Balemi and Negisho, 2012; Yu et al., 2019). This leads to a huge demand for chemical phosphate fertilizers. According to the report of the Food and Agriculture Organization of the United Nations, the world’s demand for phosphate fertilizer has reached 45.86 million tons in 2020.^1^ However, the utilization rate of chemical phosphate fertilizers is as low as 10–25% (Roberts and Johnston, 2015). Moreover, excessive chemical phosphate fertilizer would result in soil nutrition imbalance, heavy metal accumulation, and water eutrophication, meanwhile the production of chemical phosphate fertilizer often causes some other environmental pollution (Huang et al., 2017; Park et al., 2021). Therefore, developing methods to reduce the demand for chemical phosphate fertilizers and improve the bioavailability of soil TP is of significance to both agricultural production and environmental protection.

Microorganisms play an important role in the biogeochemical cycle. Among them, phosphate-solubilizing bacteria (PSB) can degrade insoluble inorganic and organic phosphorus into AP, which would then be easily absorbed and utilizated by plants (Granada et al., 2018; Wei et al., 2018; Parastesh et al., 2019; Bi et al., 2020). The previous researches screened out some PSB, which showed good P-solubilizing ability (115–716 mg/L) under laboratory culture conditions (Yu et al., 2019; Kaur and Kaur, 2020). However, it was reported that, in about 70% of the experiments, PSB did not play a direct role in supplementing soil AP for plants (Gyaneshwar et al., 2002). Considering possible effects of the different organic matter, pH, and other characteristics of the soil on the colonization and function of PSB, it is very necessary to develop suitable PSB for different types of soil.

Sedimentary rock covers 75–80% of the Earth’s crust, forming parent materials for a large majority of soils (Wilkinson et al., 2009). In the Sichuan Basin, China, more than 70% of the hills (approximately 5.0 × 10^10^ m^2^) are covered by barren rocky soil, a kind of weathered shale. Rocky soil has high potential for agricultural utilization (Manning and Theodoro, 2018). However, the AP in the rocky soil is very deficient, moreover, in which chemical phosphate fertilizer is more easily washed away and cause serious environmental pollution (Huang et al., 2017). There have been some PSB fertilizers for arable soil, but there are few studies on PSB suitable for the barren rocky soil.

The unclear mechanism of PSB is one of the important factors blocking the application of PSB (Elhaissoufi et al., 2021). Normally, direct oxidation of glucose to gluconic acid is thought to be the main P-solubilizing way in Gram-negative bacteria (Sashidhar and Podile, 2010; Luduena et al., 2018). Whereas, other studies found that pyruvate, lactic acid, succinic acid, and citric acid were the main organic acids secreted by PSB when solubilizing P (Li et al., 2017; Zeng et al., 2017). Furthermore, there is controversy regarding the effect of P availability on the P-solubilizing ability of PSB (Zeng et al., 2017). In addition, there is few systematic study on how PSB respond to P-deficiency and synthesize and secrete organic acids (Zeng et al., 2017). Fortunately, genomics, transcriptome, metabolomics, and other omics technologies have recently provided powerful methods to shed light on the P-solubilizing mechanism and application research.

The main objectives of this study were: (i) to screen highly efficient PSB suitable for the barren rocky soil, (ii) to explore the P-solubilizing mechanism at the level of genome, transcriptome, and metabolomics, and (iii) to verify the application effect of the PSB on the barren rocky soil.

## Materials and Methods

### Screening for PSB

Sweetpotato rhizosphere rocky soil samples were collected at the Yingxi experimental base of the Nanchong Academy of Agricultural Sciences, Sichuan, China (30°52'N, 106°02'E) using the methods described in a previous study (Ding et al., 2020). The soil type is purplish soils according to classification and codes for Chinese soil (GB/T 17296-2009). Soil samples (2.0 g) and sterile water (30 ml) were mixed to obtain a bacterial suspension. Bacterial strains were cultured and purified on Luria-Bertani medium according to the dilution coating method and streak plate method (Sanders, 2012).

The purified strains were inoculated on modified PKOC2 medium with sterilized toothpicks (Li et al., 2019), and the plates were inverted and incubated in a 28°C incubator. The strains with P-solubilizing circles were selected for re-screening. Holes with a diameter of 6.00 mm were punched in the screening medium. Next, 100 μl of the cultured bacterial solution with the same OD_600_ value were inoculated into the holes. After 7 days of incubation in a 28°C incubator, the diameter of the P-solubilizing circle was measured.

About 2 mm of cultured bacterial solution with the same OD_600_ value were inoculated into a 100 ml triangle flask containing 30 ml of Monkina inorganic P culture medium (Yh, 2014), with three replicates per strain. After incubation at 28°C and 150 rpm in a shaking incubator for 7 days, the pH of the medium was determined using a pH meter (PHS-3C, Fangzhou, China), and the AP of the medium was determined using the molybdenum blue method (Murphy and Riley, 1962).

### Identification of PSB

For six strains of PSB (BP10, BP11, BP23, FP2, FP12, and FP16), 2 ml of the bacterial solution were used for 16S rRNA gene sequencing. The genomic DNA were extracted using the SDS method (Natarajan et al., 2016). The amplified 16S rRNA genes were obtained with specific primers (27F: 5'-AGTTTGATCMTGGCTCAG-3'; 1492R: 5'-GGTTACCTTGTTACGACTT-3'), and sequenced using the Sanger sequencing platform (Chen et al., 2015). The 16S rRNA gene sequences were aligned to the NCBI^2^ database to search for the species with the highest similarity, wherein the selection “16S ribosomal RNA (Bacteria and Archaea)” was made. The EZBioCloud platform was further used for strain identification (Yoon et al., 2017a).

### Phosphate-Solubilizing Profile of PSB

To explore the P-solubilizing mechanism, PSB FP12, the efficient PSB selected from this study, was cultured in four kinds of media with different P sources. Four media were used as PKOC2 medium [the insoluble P medium (IPM)], PKOC2 medium with the replace of Ca_3_(PO_4_)_2_ with 3.02 g NaH_2_PO_4_•2H_2_O [the available P medium (APM)], PKOC2 medium with the addition of 3.02 g NaH_2_PO_4_•2H_2_O [the insoluble and available P medium (IAPM)], and the PKOC2 medium without Ca_3_(PO_4_)_2_ [no P medium (NPM)]. Each group had three replicates. After incubation at 28°C and 150 rpm in a shaking incubator for 7 days, the pH and AP of the medium were similarly determined as above.

### Organic Acids Targeted Metabolomics

At 1 and 2 days, as the pH and dissolved P changed largely, the solution from groups IPM and APM were sampled and filtered through a 0.2 μm filter membrane for organic acid-targeted metabolomics analysis. Each group had three replicates. The following organic acids were measured, i.e., gluconic acid, 2-keto-gluconic acid, malic acid, acetic acid, α-ketoglutaric acid, lactic acid, formic acid, citric acid, succinic acid, pyruvate, oxalic acid, and tartaric acid.

The organic acid-targeted metabolomics was performed using an Agilent liquid chromatograph (Aglient1200, United States). The liquid chromatograph column was the LAEQ-462572 Athena C18-WP 4.6 * 250 mm, with a column temperature of 30°C (van Hees et al., 1999). The detection wavelength was 210 nm (Zitouni et al., 2020). Chromatographic standard samples were used to draw standard curves to ensure that *R*^2^ > 0.99.

### Genome Sequencing of PSB FP12

The genomic DNA of PSB FP12 was extracted using the SDS method (Natarajan et al., 2016). Then the high-quality genomic DNA was sequenced using the Illumina NovaSeq sequencing platform (Illumnia, Inc., San Diego, CA, PE150). The raw sequencing data was filtered using Readfq (Version 10)^3^ with default parameters. The filtered clean data was analysed for PSB FP12 genome *de novo* assembly using SOAPdenovo (Version 2.04) with default parameters (Luo et al., 2012). Then GapCloser (Version 1.12)^4^ was used to fill gaps in the initial assembly genome. And the short contigs below 500 bp were filtered out.

GeneMarkS (Version 4.17) was used to predict the coding genes of the genome (Besemer et al., 2001). Diamond was used to align the predicted protein sequence with GO, KEGG, COG, NR, Pfam, TCDB, and Swiss-Prot (e-value ≤ 1e-5; Buchfink et al., 2015). For the alignment of each protein sequence, the match with the highest score (default identity ≥ 40%, coverage ≥ 40%) was used to select the functional annotation. A map of the circular genome was drawn with BRIG (Version 0.95; Alikhan et al., 2011). The Average Nucleotide Identity (ANI) of the genome of PSB FP12 and related species was calculated using the ANI Calculator (Yoon et al., 2017b).

### Transcriptome of PSB FP12

In order to study the transcriptional expression changes of PSB FP12 in a P-deficient environment, we analyzed the transcriptome of PSB FP12 in the insoluble P medium (IPM group) and the available P medium (APM group). At 1 and 2 days, the solution from groups IPM and APM were sampled, and each group was sampled in triplicate. The RNA of PSB FP12 was extracted using the TRIZOL method (Rio et al., 2010). Qualified libraries were sequenced on the Illumina Novaseq sequencing platform using the paired-end sequencing method (PE150). FastQC_v0.11.3 was used for quality control of the raw sequencing data. The filtered sequence was aligned with the rRNA database using Bowtie2 to remove the rRNA sequence (Langmead and Salzberg, 2012).

Qualified sequencing data were aligned to the PSB FP12 genome using Hisat2 (Kim et al., 2015), and the count value and the Reads Per Kilobase per Million mapped reads (RPKM) values of the genes were calculated using HTseq-count and AWK script (Anders et al., 2015). EdgeR was used to calculate the differentially expressed genes [*p* < 0.05 and |Log_2_ (fold change)| > 1; Robinson et al., 2009]. Gene Ontology (GO) classification and enrichment analysis of differentially expressed genes was performed on the WEGO 2.0 platform (Ye et al., 2018). KEGG enrichment analysis of differentially expressed genes was performed based on the KEGG database.^5^

### Treating the Barren Rocky Soil With PSB

To verify the application effects of those selected PSB on the barren rocky soil, a series of experiments were performed. The barren rocky soil was collected at the Yingxi, Nanchong, Sichuan, China (30°52′N, 106°02′E). The soil type is purplish soils according to Classification and codes for Chinese soil (GB/T 17296-2009; Supplementary Figure S1). The barren rocky soil properties were showed in Supplementary Table S1. About 1 ml of single PSB (BP10, BP11, BP23, FP2, FP12, or FP16; approximately 10^9^ CFU/ml) or the mixed PSB (MB group) were added to 10 g of sterilized barren rocky soil. Around 1 ml sterile water without PSB was added in the control (CK) group. After 10 days of incubation in a 28°C incubator, the soil pH and AP were measured using the methods described in a previous study (Ding et al., 2020).

### Addition of Sweetpotato Root Exudates

One of the main factors limiting the colonization of PSB in the barren rocky soil is the lack of organic matter. Plant root exudates are important sources of soil organic matter, and plants can affect the colonization of rhizosphere microorganisms through root exudates (Sasse et al., 2018). Therefore, the effect of plant root exudates was also considered when developing PSB suitable for the barren rocky soil.

Sweetpotato root exudates were collected according to methods described previously (Ding et al., 2020). BP10, BP11, BP23, FP2, FP12, and FP16 PSB (approximately 10^9^ CFU/ml) were mixed in equal proportions and then centrifuged. The supernatant was discarded, and the remaining pellet was resuspended in sterile water. Around 10 ml of the resuspended PSB was added to 100 g of sterilized barren rocky soil, then 10 ml of sweetpotato root exudates was added as a treatment group (BR group), and 10 ml of sterile water was added as a control group (BW group). Meanwhile, 20 ml of sterile water was added to 100 g of sterilized soil, without PSB, as another control (BCK group). After 10 days of incubation in a 28°C incubator, the soil pH and AP were measured using the methods described in a previous study (Ding et al., 2020).

### Pot Experiments

A greenhouse pot experiment was performed in a greenhouse (30°63′N, 104°07′E) at Chengdu Institute of Biology, Chinese Academy of Sciences. A strain of sweetpotato [*Ipomoea batatas* (L.) Lam.] cultivar Nanshu 88 was planted in a pot (high, 26.5 cm; diameter 24.0 cm) containing 6.5 kg of barren rocky soil. In the BP group, 75 ml of mixed PSB (approximately 10^9^ CFU/ml) was applied to each pot; in the CK group, 75 ml of sterile water without PSB was applied. Each group had eight pots. The greenhouse temperature was 25°C.

The field pot experiment was performed at the Yingxi experimental base (30°52′N, 106°02′E), and designed same as in the greenhouse pot experiment. During the experiment, the local monthly precipitation was 64–180 mm, and the mean temperature was 18.5–28.4°C.

At 100 days, the sweetpotato and soil in the greenhouse and field pot experiments were weighed and sampled. The P and potassium (K) content of sweetpotato roots and vines were measured using an elemental content analyzer with inductively coupled plasma optical emission spectrometry (ICP-OES; Optima 8300, PerkinElmer, United States). The nitrogen (N) content of sweetpotato roots and vines, as well as the pH, total carbon (TC), total nitrogen (TN), and AP of soil were measured using the methods described in a previous study (Ding et al., 2020).

### Statistics Analysis

Significant differences between samples were evaluated using Tukey’s honest significant difference test on R (version 3.5.0). Spearman’s correlation coefficients between AP and pH were also calculated using R (version 3.5.0).

## Results

### Screening and Identification of PSB

After preliminary screening, 26 bacterial strains that grew well on the screening media and had obvious P-solubilizing circles were selected for re-screening. These 26 PSB strains were inoculated into the round holes of the screening media to observe the P-solubilizing circle (Supplementary Figure S2A). At 7 days, the P-solubilizing circle of BP11 was the largest one, with a diameter of 25.75 mm, and the ratio of the P-solubilizing circle to the diameter of the round hole (D/d) was 4.29. In addition, the P-solubilizing circle of PSB FP12 was also observed with a large diameter of 23.75 mm and D/d of 3.96 (Supplementary Table S2).

**Figure 1:**
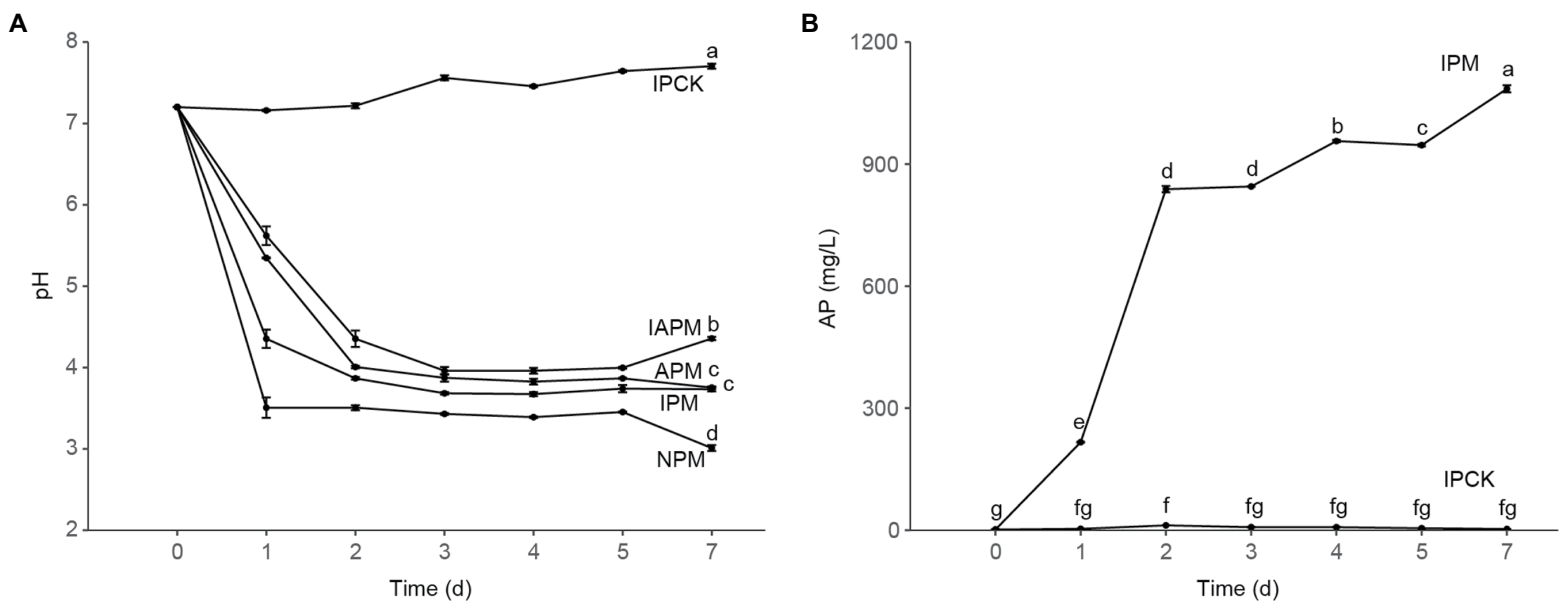
The pH and solubilized available phosphorus (AP). **(A)** The change of pH in mediums with different phosphorus sources. Phosphate-solubilizing bacteria (PSB) FP12 was cultured in four kinds of media, i.e., insoluble phosphorus medium (IPM); available phosphorus medium (APM); insoluble and available phosphorus medium (IAPM); medium without phosphorus (NPM). IPCK is insoluble phosphorus medium without PSB. **(B)** The AP dissolved by phosphate-solubilizing bacteria FP12 in IPM group. Different letters denote significant difference from a Tukey’s HSD test (*p* < 0.05).

These 26 PSB strains were inoculated and screened in the liquid media, and the AP content was measured at 7 days (Supplementary Figure S2B). Most strains showed significantly higher values of AP than the control group. The AP of FP12 reached 744.00 mg/L at 7 days, which was significantly higher than that of other strains (*p* < 0.001; Supplementary Figure S2B; Supplementary Table S3) and was further optimized to 1,085.00 mg/L (Figure 1).

Combined with the re-screening results of the P-solubilizing ability on plate and shake flask, we finally selected BP10, BP11, BP23, FP2, FP12, and FP16 for further analysis. Through 16S rRNA sequencing, PSB BP10, BP11, BP23, FP2, FP12, and FP16 were identified as *Stenotrophomonas maltophilia*, *Achromobacter xylosoxidans*, *Achromobacter xylosoxidans*, *Stenotrophomonas maltophilia*, *Ochrobactrum haematophilum*, and *Cellulosimicrobium cellulans*, respectively (Supplementary Table S4).

### The Relationship Between pH and Solubilized AP

To study whether the P-solubilizing mechanism is related to acid production, the relationship between solubilized AP and pH was analyzed. The correlation analysis showed that there was a significant negative correlation between the solubilized AP and pH; Spearman *ρ* was −0.92 (*p* < 2.2e–16). The trend analysis showed that the solubilized AP and pH correlated well in line with the logarithmic function: pH = −0.565 ln (AP) + 7.7973, *R*^2^ = 0.8801.

As shown in Figure 1A, PSB FP12 decreased the pH of the NPM and IPM groups rapidly, which was significantly lower than that of the APM group. This result indicated that the AP-deficient environment could enhance the acidification of PSB FP12, so as to decrease the pH of the medium. We also found that the pH of the IAPM and APM group media were significantly higher than those of the IPM and NPM group media (Figure 1A), indicating that AP might weaken the acidification of PSB FP12.

Along with the decrease of pH, PSB FP12 rapidly increased AP in the IPM group. The AP of the IPM group significantly increased to 216.50 mg/L at 1 days, 838.33 mg/L at 2 days, and 1,085.00 mg/L at 7 days, respectively. The AP of the IPM group was always significantly higher than that of the IPCK group (Figure 1B).

### Organic Acid-Targeted Metabolomics of PSB FP12

**Figure 2:**
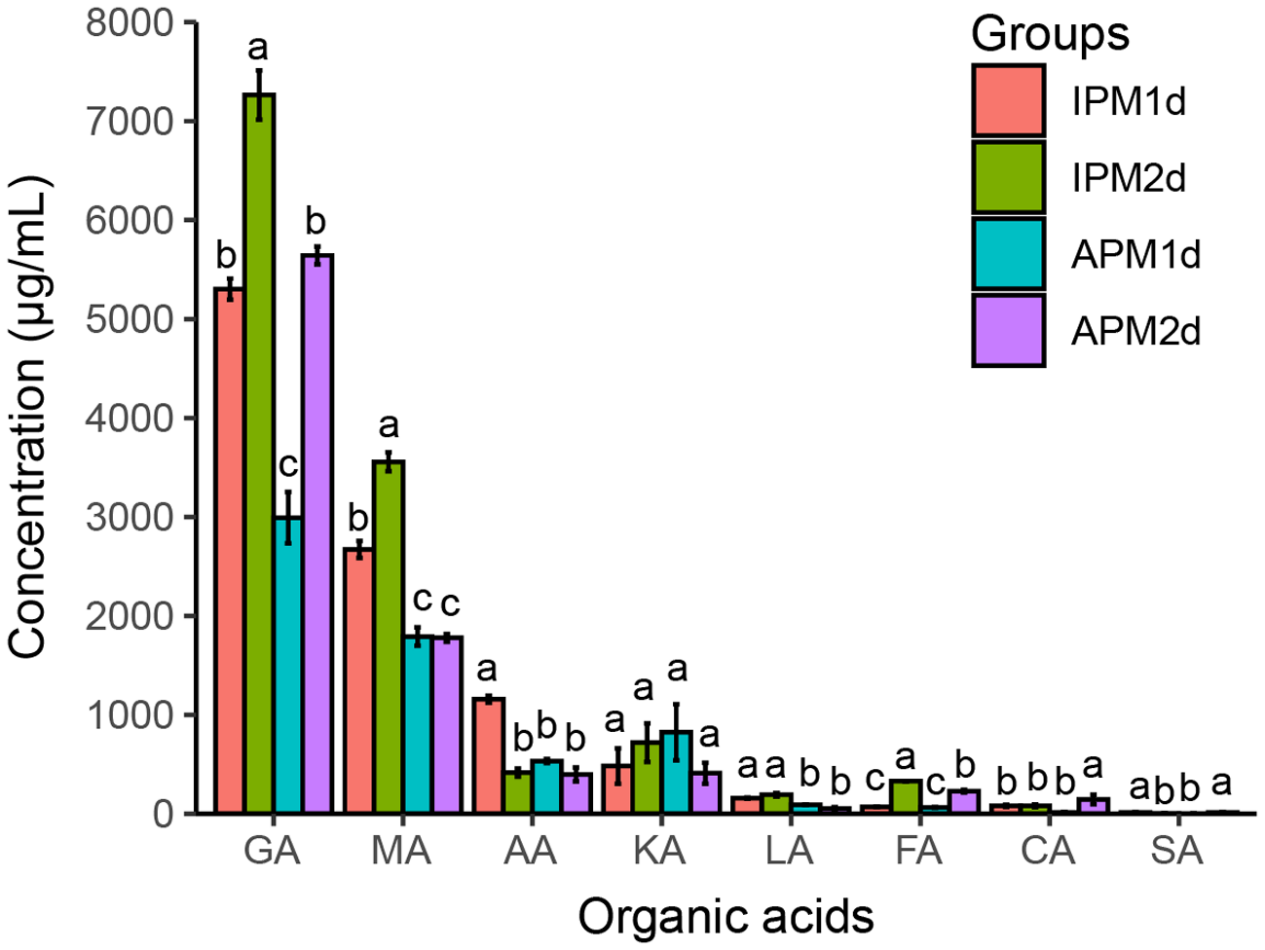
The concentration of organic acids in IPM and APM groups. IPM1d and IPM2d represent 1 day and 2 days after inoculation of phosphate-solubilizing bacteria FP12 in the insoluble phosphorus medium, respectively. APM 1d and APM 2d represent 1 day and 2 days after inoculation of phosphate-solubilizing bacteria FP12 in the available phosphorus medium, respectively. GA, gluconic acid; MA, malic acid; AA, acetic acid; KA, α-ketoglutaric acid; LA, lactic acid; FA, formic acid; CA, citric acid; and SA, succinic acid. Different letters denote significant difference from a Tukey’s HSD test (*p* < 0.05).

The organic acid concentrations of the IPM and APM groups at 1 and 2 days were measured. Gluconic acid, malic acid, acetic acid, α-ketoglutaric acid, lactic acid, formic acid, citric acid, and succinic acid were detected, while pyruvate, oxalic acid, tartaric acid, and 2-keto-gluconic acid were not detected. Among them, gluconic acid showed the highest content, followed by malic acid, acetic acid, and α-ketoglutaric acid (Figure 2).

The concentrations of organic acids in the IPM group were generally higher than these in the APM group. The concentrations of gluconic acid in the IPM group were 5,302.04 and 7,263.62 μg/ml at 1 and 2 days, respectively, which were significantly higher than these (2,992.47 μg/ml at 1 days, 5,642.76 μg/ml at 2 days) in the APM group. The concentrations of malic acid in the IPM group were 2,671.39 and 3,556.95 μg/ml at 1 and 2 days, respectively, which were significantly higher than these (1,791.03 μg/ml at 1 days, 1,778.81 μg/ml at 2 days) in the APM group. The concentrations of acetic acid and lactic acid in the IPM group were also significantly higher than these in the APM group (Figure 2).

### Genome of PSB FP12

**Figure 3:**
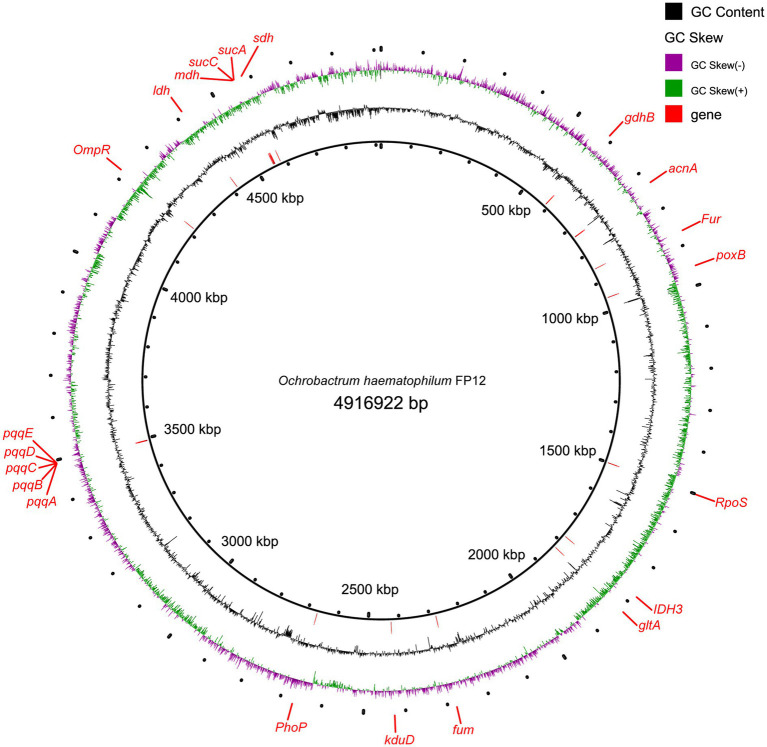
The genome of *Ochrobactrum haematophilum* FP12 and genes related to phosphate-solubilizing.

The final assembled genome size was 4.92 Mb with a scaffold N50 of 0.32 Mb, and a GC content of 57.05%. Total 4,714 genes were predicted in the PSB FP12 genome, with a total length of 4.27 Mb, accounting for 86.92% of the genome (Figure 3). While compared with the published genomes, PSB FP12 genome showed the highest ANI of 97.84% with the *O. haematophilum* strain FI11154 genome (Supplementary Table S5), which indicated that they are same species.

In the PSB FP12 genome, genes for the main enzymes and their coenzymes involved in gluconic acid and 2-keto-gluconic acid synthesis were found to be: glucose dehydrogenase (GDH) gene *gdhB* (Quinoprotein glucose dehydrogenase, FP12_GM000535), pyrroloquinoline quinone (PQQ) synthesis protein series genes pqqA-E (FP12_GM003319, FP12_GM003320, FP12_GM003321, FP12_GM003322, and FP12_GM003323), and gluconate dehydrogenase gene *kduD* (FP12_GM002270; Figure 3).

In addition, the indole acetic acid (IAA) synthetic gene [aldehyde dehydrogenase (*ALDH*)] and siderophores biosynthesis genes were also identified in the PSB FP12 genome.

### Transcriptome of PSB FP12

**Figure 4:**
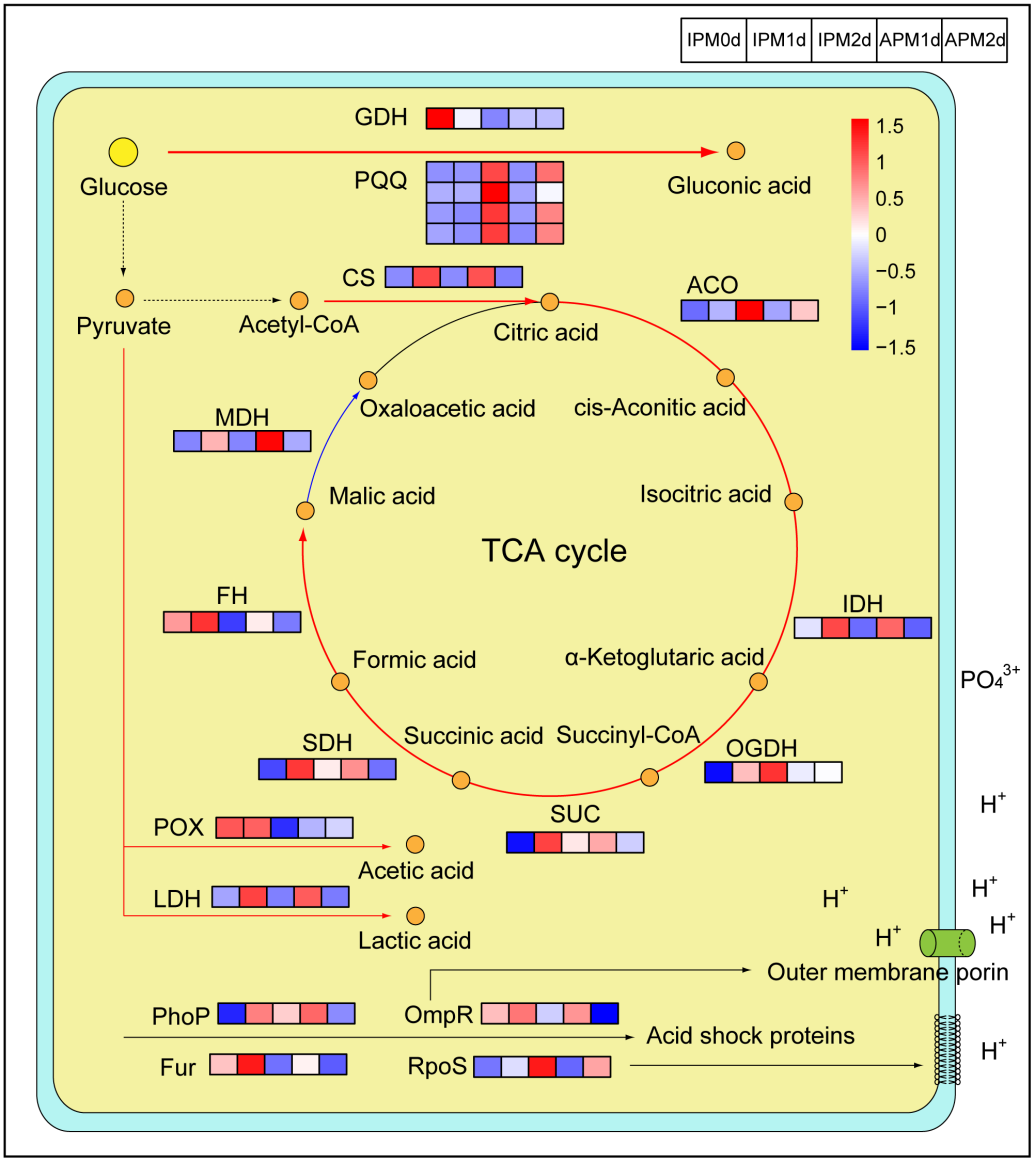
The expression level of genes related to organic acids metabolism and acid tolerance. IPM0d, IPM1d and IPM2d represent 0 day, 1 day, and 2 days after inoculation of phosphate-solubilizing bacteria FP12 in the insoluble phosphorus medium, respectively. APM 1d and APM 2d represent 1 day and 2 days after inoculation of phosphate-solubilizing bacteria FP12 in the available phosphorus medium, respectively. Red represents high expression, and blue represents low expression. GDH, glucose dehydrogenase; PQQ, pyrroloquinoline quinone; CS, citrate synthase; ACO, aconitic hydratase; IDH, isocitrate dehydrogenase; OGDH, α-ketoglutarate dehydrogenase; SUC, succinyl-CoA synthetase; SDH, succinate dehydrogenase; FH, fumarate hydratase; MDH, malate dehydrogenase; POX, pyruvate dehydrogenase; and LDH, L-lactate dehydrogenase.

The GO enrichment results showed that the differentially expressed genes between the IPM and APM groups may be related to the response of the PSB to the AP-deficient environment, the regulation of their own metabolism, and the synthesis and secretion of organic acids (Supplementary Figures S3, S4). KEGG enrichment results showed that the gene expression of the organic acid synthesis pathways, such as the gluconic acid synthesis pathway, the TCA cycle, and the pyruvate metabolic pathway, were upregulated in the AP-deficient environment (Figure 4; Supplementary Table S6).

In our study, the expression of the GDH gene *gdhB* was significantly higher than that in the APM group at 1 days (*p* < 0.0001), and the expression levels of the PQQ synthesis protein genes, i.e., *pqqB*, *pqqC*, *pqqD*, and *pqqE* in the IP group were higher than those in the APM group (Figure 4).

Transcriptome analysis revealed that the expression of genes related to the TCA cycle in the IPM group was generally higher than those in the APM group, such as citrate synthase (CS), aconitic hydratase (ACO), isocitrate dehydrogenase (IDH), α-ketoglutarate dehydrogenase (OGDH), succinyl-CoA synthetase (SUC), succinate dehydrogenase (SDH), and fumarate hydratase (FH) genes (Figure 4). However, the expression level of the malate dehydrogenase (MDH) gene, *mdh*, in the IPM group was 671.88 RPKM at 1 days, which was lower than that (1088.42 RPKM) in the APM group, and this gene also showed lower expression levels in the IPM group (217.49 RPKM) than in the APM group (309.99 RPKM) at 2 days (Figure 4).

The expression of pyruvate dehydrogenase (POX) gene in the IPM group was significantly higher than that in the APM group (*p* < 0.01; Figure 4). The expression of the L-lactate dehydrogenase (LDH) gene in the IPM group was significantly higher than that in the APM group (*p* < 0.01; Figure 4).

Bacteria induce an acid tolerance response by producing acid shock proteins and changing cell membrane fluidity. We found that the expression levels of these regulatory proteins in the IPM group were higher than those in the APM group (Figure 4). The expression levels of RpoS in IPM1d and IPM2d were 2682.65 RPKM and 5549.95 RPKM, respectively, which were significantly higher than those in APM group (1371.87 RPKM and 3997.41 RPKM; *p* < 0.01). The expression level of OmpR in IPM1d was 584.40 RPKM, which was higher than APM1d (556.62 RPKM); and the expression level of OmpR in IPM2d was 414.22 RPKM, which was significantly higher than that of APM1d (223.08 RPKM; *p* < 0.05; Figure 4).

### Treating Barren Rocky Soil With PSB

**Figure 5:**
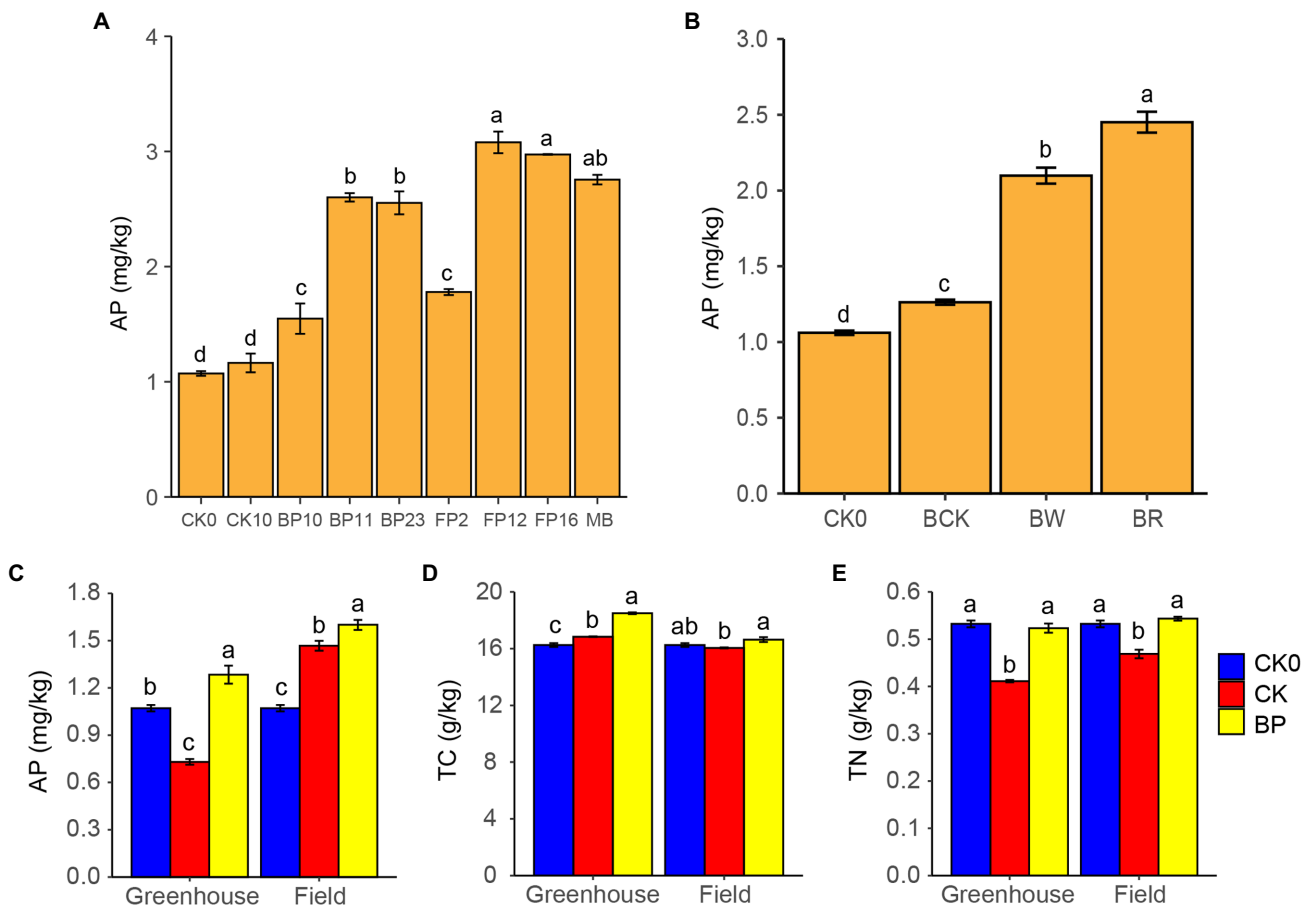
Phosphate-solubilizing bacteria application potential. **(A)** AP in the barren rocky soil after treatment by phosphate-solubilizing bacteria, CK0, control group 0 day; CK10, control group 10 days; MB, mixed phosphate-solubilizing bacteria. **(B)** Effect of sweetpotato root exudates on phosphate-solubilizing, CK0, control group 0 day; BCK, control group without mixed phosphate-solubilizing bacteria 10 days; BW, mixed phosphate-solubilizing bacteria with sterile water; BR, mixed phosphate-solubilizing bacteria with sweetpotato root exudates. **(C-E)** Soil properties in greenhouse and field pot experiments, AP, available phosphorus; TC, total carbon; TN, total nitrogen; CK0 group, control group 0 day; CK group, control group without phosphate-solubilizing bacteria 100 days; and BP group, mixed phosphate-solubilizing bacteria was applied 100 days. Different letters denote significant difference from a Tukey’s HSD test (*p* < 0.05).

The six strains of PSB and mixed PSB significantly increased the AP in the barren rocky soil and their AP contents were significantly higher than those in the control group (Figure 5A). Among them, PSB FP12 showed the highest P-solubilizing efficiency, which significantly increased the soil AP from 1.07 to 3.08 mg/kg. The mixed PSB also significantly increased the soil AP to 2.76 mg/kg (Figure 5A). Meanwhile, we found that PSB treatment significantly reduced soil pH. The pH of BP10, BP11, BP23, FP2, FP12, FP16, and the mixed PSB groups significantly reduced from 8.54 to 8.17, 8.10, 8.19, 8.06, 8.17, 8.16, and 8.13, respectively.

### Effects of Sweetpotato Root Exudates on P Solubilization

The addition of sweetpotato root exudates significantly improved the P-solubilizing effect of the PSB (Figure 5B). The AP in the BR group increased from 1.07 to 2.45 mg/kg, which was significantly higher than that (2.10 mg/kg) in the BW group. The AP in the BR and BW groups was significantly higher than that in the BCK group (1.26 mg/kg; Figure 5B). Meanwhile, we found that the pH of barren rocky soil in the BR group decreased significantly from 8.54 to 8.35, which was significantly lower than that in the BW group (8.56).

### Greenhouse and Field Pot Experiments

The application of PSB significantly increased the AP in the barren rocky soil. In the greenhouse and field pot experiment, AP in the barren rocky soil increased significantly from 1.07 to 1.28 mg/kg and 1.63 mg/kg in the BP group, respectively, which was both significantly higher than those (0.73 and 1.47 mg/kg) in the CK group (*p* < 0.05; Figure 5C). In addition, if the P absorbed by sweetpotato is taken into account (4.52 and 7.10 mg P per sweetpotato in the greenhouse and field pot experiment, respectively), the PSB actually releases more P.

The application of PSB also affected the barren rocky soil properties such as pH, TC, and TN. Applying PSB decreased the pH of the barren rocky soil, which is beneficial to reduce alkalization of barren rocky soil. Meanwhile, applying PSB increased the TC and TN of barren rocky soil (Figures 5D,E).

Applying PSB increased the tuberous roots and vines yields, as well as N, P, and K content in sweetpotato. In the greenhouse and field pot experiment, the yields of tuberous roots of sweetpotato were 26.08 and 36.43 g in the BP group, respectively, which were significantly higher than those (16.21 and 29.38 g) in the CK group (*p* < 0.05). Meanwhile, applying PSB increased the ratio of tuberous roots to total biomass of sweetpotato. Moreover, applying PSB even significantly increased the N, P, and K content in the sweetpotato roots and vines (Table 1; Supplementary Figure S5).

**Table 1:** Physiological data of sweetpotato in greenhouse and field pot experiments.

		Tuberous roots (g)	Fibrous roots (g)	Vines (g)	Roots (g/kg)			Vines (g/kg)		
					N	P	K	N	P	K
Greenhouse pot	CK	16.21 ± 5.95	2.78 ± 0.98	14.26 ± 4.02	4.65 ± 0.02	0.31 ± 0.01	15.30 ± 0.01	8.84 ± 0.01	0.57 ± 0.00	17.64 ± 0.02
	BP	26.08 ± 4.45^*^	3.93 ± 1.94	17.89 ± 3.37	7.41 ± 0.00^*^	0.38 ± 0.00^*^	16.05 ± 0.03^*^	12.84 ± 0.01^*^	0.61 ± 0.00^*^	18.74 ± 0.01^*^
Field pot	CK	29.38 ± 6.23	11.37 ± 2.68	26.88 ± 6.51	4.97 ± 0.01	0.54 ± 0.00	16.57 ± 0.01	7.83 ± 0.01	0.61 ± 0.00	17.40 ± 0.01
	BP	36.43 ± 4.76^*^	10.58 ± 5.02	31.25 ± 6.94	7.54 ± 0.01^*^	0.57 ± 0.00^*^	16.91 ± 0.01^*^	11.36 ± 0.02^*^	0.63 ± 0.00	17.91 ± 0.03^*^

CK group, control group without phosphate-solubilizing bacteria 100 days; BP group, mixed phosphate-solubilizing bacteria was applied 100 days.

* *p* < 0.05.

## Discussion

Our previous study found that sweetpotato grew well in AP-deficient rocky soil, and some PSB may be present in the sweetpotato rhizosphere (Ding et al., 2020). In this study, we screened six highly efficient PSB from sweetpotato rhizosphere rocky soil. Among them, *O. haematophilum* FP12 showed the highest P-solubilizing ability of 1,085.00 mg/L at 7 days. To our knowledge, it showed higher P-solubilizing ability than most of previously reported PSB (Chen et al., 2006; Sarikhani et al., 2019; Kaur and Kaur, 2020).

### Multi-Omics Reveal the Highly Efficient P-Solubilizing Mechanism of PSB FP12

Organic acid secretion is an important P-solubilizing way in PSB (Zeng et al., 2016). We found that the concentrations of organic acids in the IPM group were generally higher than those in the APM group, especially gluconic acid and malic acid, whose concentrations in the IPM group were 1.29–2.00 times higher than those in the APM group (Figure 2). These results may explain why the pH of the IPM group was lower than that of the APM group (Figure 1A). Thus, AP-deficiency could promote the secretion of organic acid by PSB, while AP inhibited it. This result is consistent with the previous finding (Zeng et al., 2017).

Direct oxidation of glucose to gluconic acid is thought to be the main P-solubilizing way in Gram-negative bacteria (Sashidhar and Podile, 2010; Luduena et al., 2018). We found that gluconic acid was the most prevalent organic acid in the media (Figure 2), which plays a major role in the rapid P-solubilization by PSB FP12. Whereas, a study in the PSB *Burkholderia multivorans* WS-FJ9 found that pyruvate was the main organic acid secreted when solubilizing P (Zeng et al., 2017). Another study in the PSB *Enterobacter cloacae* RW8 found that lactic acid, succinic acid, and citric acid were the main organic acids secreted (Li et al., 2017). These results showed that different PSB would secrete the varied types and concentrations of organic acids to solubilize P.

The genome contains the genetic information that allows bacteria to function. Genes related to various organic acid synthesis, acid shock regulatory proteins (RpoS, OmpR, PhoP, and Fur), acid/alkaline phosphatase, IAA synthesis, and siderophores biosynthesis were identified in the PSB FP12 genome (Figure 3). The acid/alkaline phosphatase suggested that PSB FP12 may degrade organic P. And the IAA synthesis and siderophores biosynthesis genes suggested that PSB FP12 may promote plant growth through producing IAA and siderophores, in addition to solubilizing P (Luduena et al., 2018).

A previous study has illustrated the important role of gluconic acid synthesis in P-solubilizing (Farhat et al., 2013). In our study, the expression of the GDH genes in the IPM group were higher than that in the APM group (Figure 4), which may result to the higher gluconic acid concentration in the IPM group (Figure 2). Our results differ from those in *B. multivorans* WS-FJ9, whose expression of gluconic acid synthesis genes are not affected by P availability (Zeng et al., 2017). This difference may be due to the different P-solubilizing mechanisms among PSB strains. For example, the PSB *B. multivorans* WS-FJ9 mainly secreted pyruvate to solubilize P, not gluconic acid (Zeng et al., 2017).

The TCA cycle produces a variety of organic acids, such as citric acid, α-ketoglutaric acid, succinic acid, and malic acid (Akram, 2014; Vuoristo et al., 2016). Transcriptome analysis revealed that the expression of genes related to the TCA cycle in the IPM group was generally higher than that in the APM group, excepting the MDH gene (Figure 4). MDH catalyzes malic acid to oxaloacetate (Sutherland and McAlisterhenn, 1985). Thus, low expression of MDH gene may be the reason why malic acid concentration in the IPM group was higher than that in the APM group (Figure 2). Pyruvate can be converted to organic acids such as acetic acid and lactic acid through the pyruvate metabolic pathway (Melo et al., 2018). Transcriptome analysis revealed that the expression of related genes (POX, LDH) was higher than that in the APM group (Figure 4), which might induce the higher acetic acid and lactic acid concentration in the IPM group (Figure 2).

Bacteria induce an acid tolerance response by producing acid shock proteins and changing cell membrane fluidity. Regulatory proteins such as RpoS, OmpR, PhoP, and Fur can affect the production of acid shock proteins (Hall and Foster, 1996). In addition, RpoS reduces cell membrane fluidity (Spector and Kenyon, 2012), and OmpR directly affects the outer membrane porin OmpC and OmpF to respond to external acids (Csonka and Hanson, 1991). We found that the expression levels of these regulatory proteins in the IPM group were significantly higher than those in the APM group (*p* < 0.05; Figure 4). These results indicated that PSB FP12 enhanced its acid tolerance response in the IPM group, which helped it survive in an acidic environment.

Therefore, in an AP-deficient environment, PSB FP12 upregulated the expression of genes in the organic acid synthesis pathway and secreted more organic acids such as gluconic acid, malic acid, and acetic acid (Figures 2, 4). The expression patterns of related genes and the secretion of organic acids in PSB FP12 were different from some of those previously reported PSB, indicating that different PSB have various P-solubilizing mechanisms.

### The Screened PSB Significantly Increased the AP in the Barren Rocky Soil From the Laboratory to the Field

The application of PSB is affected by many factors, such as soil properties, rhizosphere colonization, and the local climate (Gyaneshwar et al., 2002). A previous study found that P-solubilizing microorganisms did not play a direct role in supplementing soil AP for plants in about 70% of the experiments (Gyaneshwar et al., 2002). Therefore, we further studied the application effect of PSB through experiments such as treating the barren rocky soil with PSB and adding sweetpotato root exudates, as well as greenhouse potting and field potting (Supplementary Table S7). The barren rocky soil experiment indicated that all of these screened PSB had P-solubilizing effects on the barren rocky soil. Considering that the composite strains have stronger adaptability to complex environments, mixed PSB was used for further experiments (Scheuerl et al., 2020).

We found that adding sweetpotato root exudates improved the P-solubilizing effect of the PSB (Figure 5B). Plant root exudates are important sources of nutrients such as carbon and N for rhizosphere microorganisms, and plants can also affect the colonization of rhizosphere microorganisms through root exudates (Sasse et al., 2018). The sweetpotato root exudates might increase the P-solubilizing effect through promoting the colonization of PSB.

The results of the greenhouse and field pot experiments were consistent. The application of PSB increased the AP, TC, and TN in the barren rocky soil (Figures 5C–E). And the P-solubilizing effect of PSB was higher than that of most of previously reported PSB (Zhu et al., 2018). In addition, based on the preliminary 16S rRNA sequencing results of our follow-up field experiment, the relative abundance of the PSB could be more than 1%, which was significantly higher than that of the control (unpublished data). Moreover, P is an essential macro element required for plants, which is especially important for early stage root development, stem strength, and yield (Satyaprakash et al., 2017). In this study, the application of PSB increased the yield of sweetpotato tuberous roots, biomass and the ratio of tuberous roots to total biomass, and the content of N, P, and K in the roots and vines (Table 1). These results indicated that in the greenhouse and field environments, the PSB could improve the barren rocky soil fertility and promote sweetpotato growth. In adition, some PSB also showed a good growth-promoting effect on a variety of crops, such as *O. haematophilum* could promote the growth of corn and tobacco (Pereira and Castro, 2014; Gao et al., 2016). It would be an interesting and valuable work to study the effect of these screened PSB, especially the FP12, on other crops in future.

Chemical phosphate fertilizers applied to the barren rocky soil is easy to be washed away and cause serious environmental pollution (Huang et al., 2017). In addition, our preliminary research found that commercialized PSB did not work well in the barren rocky soil. Here, the screened PSB can effectively solubilize P and continuously alleviate the AP-deficiency in the barren rocky soil, showing great potential in reducing the application of chemical phosphate fertilizer and the related environmental pollution. Therefore, to pave the way toward their actual application in the rocky soil, more efficient PSB would be screened and the possible interaction among these screened PSB would be studied in future. More importantly, the biosafety potential of those screened PSB, especially the *O. haematophilum* FP12, would also be carefully and thoroughly analyzed before promoting their large-scale applications in the rocky soil.

## Conclusion

We screened six strains of PSB from sweetpotato rhizosphere rocky soil. Among them, the PSB *O. haematophilum* FP12 had the best P-solubilizing ability, reaching 1,085.00 mg/L at 7 days. The assembled genome of PSB FP12 was 4.92 Mb in length, containing 4,714 genes, including P-solubilizing and plant growth-promoting genes. In AP-deficient environment, PSB FP12 upregulated the expression of organic acid synthesis genes and produced more organic acids, leading to P-solubilization. Moreover, these PSB improved the barren rocky soil fertility and promoted sweetpotato yield in the laboratory and field experiments. This study provides efficient and adaptable PSB strains for barren rocky soil, which will reduce the environmental pollution caused by excessive chemical phosphate fertilizer and promote the environment-friendly agricultural development.

## Data Availability Statement

The datasets presented in this study can be found in online repositories. The names of the repository/repositories and accession number(s) can be found at: https://www.ncbi.nlm.nih.gov/, PRJNA662158, PRJNA662170, and MW454804-MW454809.

## Author Contributions

YD: conceptualization, methodology, data curation, software, writing – original draft, and writing – review and editing. ZY: methodology, data curation, formal analysis, and writing – review and editing. YF: investigation and data curation. SH and YL: investigation. KH: resources and writing – review and editing. HZ: writing – review and editing and funding acquisition. YJ: writing – review and editing, supervision, project administration, and funding acquisition. All authors contributed to the article and approved the submitted version.

## Funding

This study was supported by the China Agriculture Research System of MOF and MARA (CARS-10-B24), the National Key R&D Program of China (2018YFF0213505), the National Natural Science Foundation of China (31770395), the Science & Technology Program of Sichuan Province (2017HH0077), and Innovation Academy for Seed Design, CAS.

## Conflict of Interest

The authors declare that the research was conducted in the absence of any commercial or financial relationships that could be construed as a potential conflict of interest.

## Publisher’s Note

All claims expressed in this article are solely those of the authors and do not necessarily represent those of their affiliated organizations, or those of the publisher, the editors and the reviewers. Any product that may be evaluated in this article, or claim that may be made by its manufacturer, is not guaranteed or endorsed by the publisher.

## Figures and Tables

**Table:** 

AP	Available phosphorus
P	Phosphorus
PSB	Phosphate-solubilizing bacteria
TP	Total phosphorus
ANI	Average Nucleotide Identity
RPKM	Reads Per Kilobase per Million mapped reads
K	Potassium
N	Nitrogen
TC	Total carbon
TN	Total nitrogen
D/d	The ratio of the P-solubilizing circle to the diameter of the round hole
PQQ	Pyrroloquinoline quinone
IAA	Indole acetic acid
ALDH	Aldehyde dehydrogenase
GDH	Glucose dehydrogenase
CS	Citrate synthase
ACO	Aconitic hydratase
IDH	Isocitrate dehydrogenase
OGDH	α-ketoglutarate dehydrogenase
SUC	Succinyl-CoA synthetase
SDH	Succinate dehydrogenase
FH	Fumarate hydratase
MDH	Malate dehydrogenase
POX	Pyruvate dehydrogenase
LDH	L-lactate dehydrogenase

## Footnotes

^1^ http://www.fao.org/

^2^ https://blast.ncbi.nlm.nih.gov/

^3^ http://github.com/cjfields/readfq

^4^ https://sourceforge.net/projects/soapdenovo2/files/GapCloser/

^5^ https://www.kegg.jp/
