# Supplementary material for: Multi-Omics Reveal the Efficient Phosphate-Solubilizing Mechanism of Bacteria on Rocky Soil
Source: Front Microbiol. 2021 Dec 9;12:761972. doi: 10.3389/fmicb.2021.761972 (PMC8696128; doi:10.3389/fmicb.2021.761972)
Supplement: Supplementary file 1 [file Data_Sheet_1.docx]

Supplementary Material

## Supplementary Figures


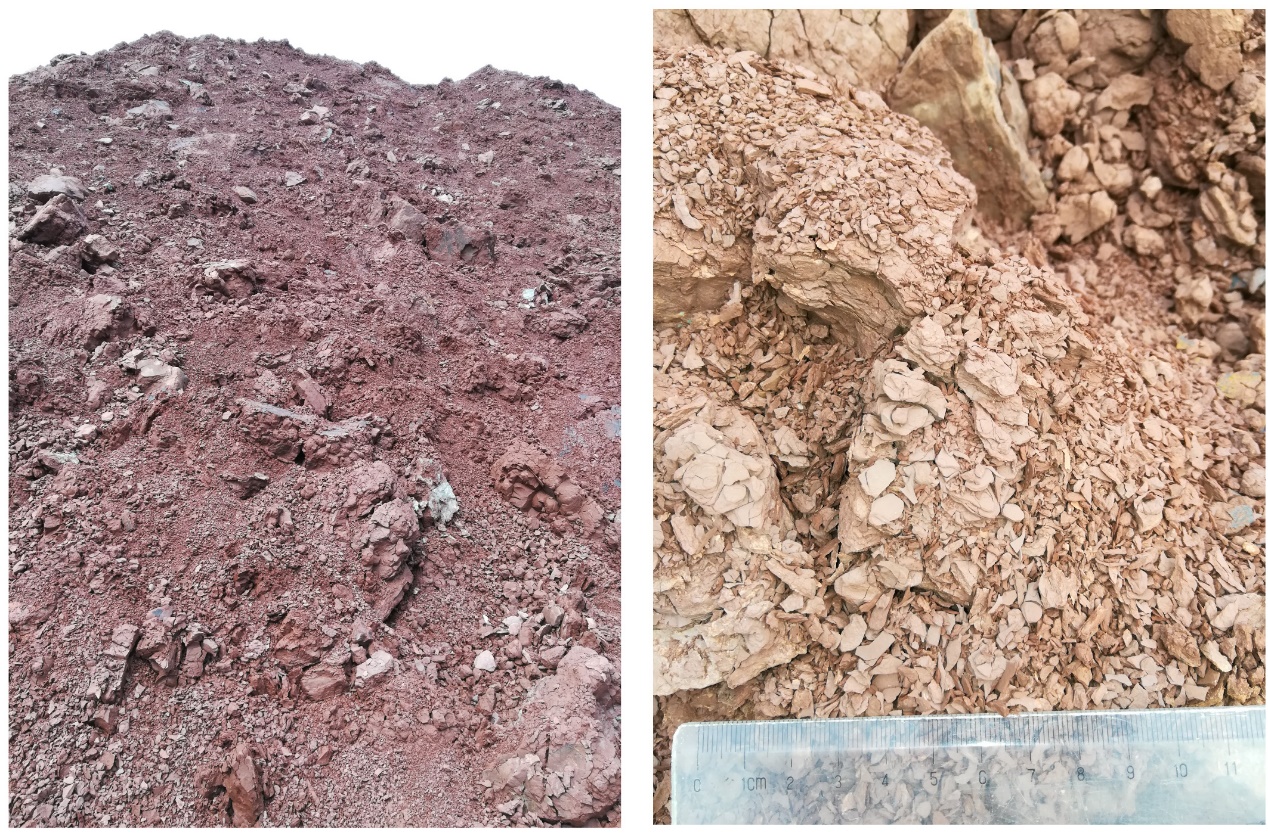


**Supplementary Figure 1.** The barren rocky soil


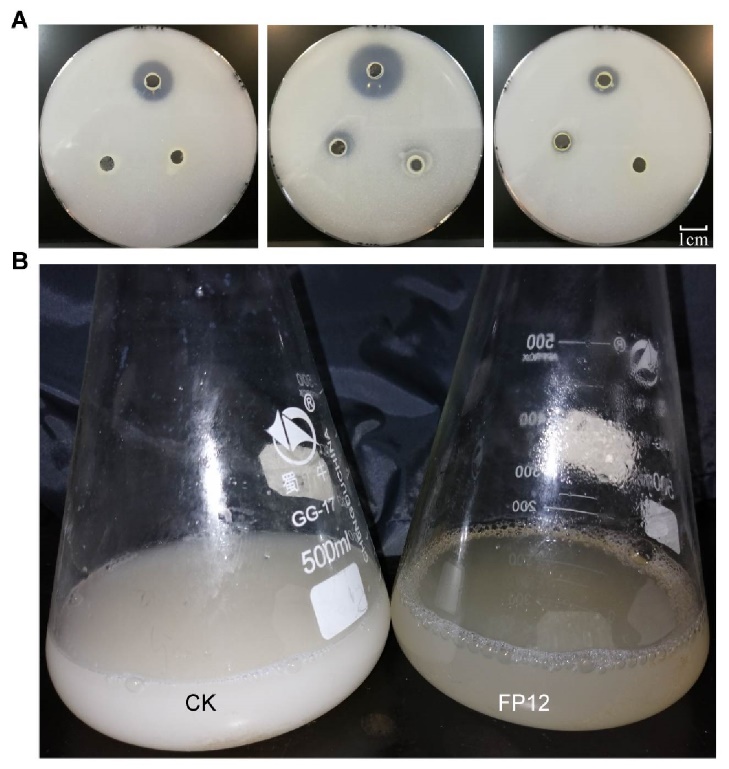


**Supplementary Figure 2.** The screening of phosphate-solubilizing bacteria

(A), the phosphate-solubilizing circle; (B), comparison of control group and phosphate-solubilizing bacteria FP12 treatment group.


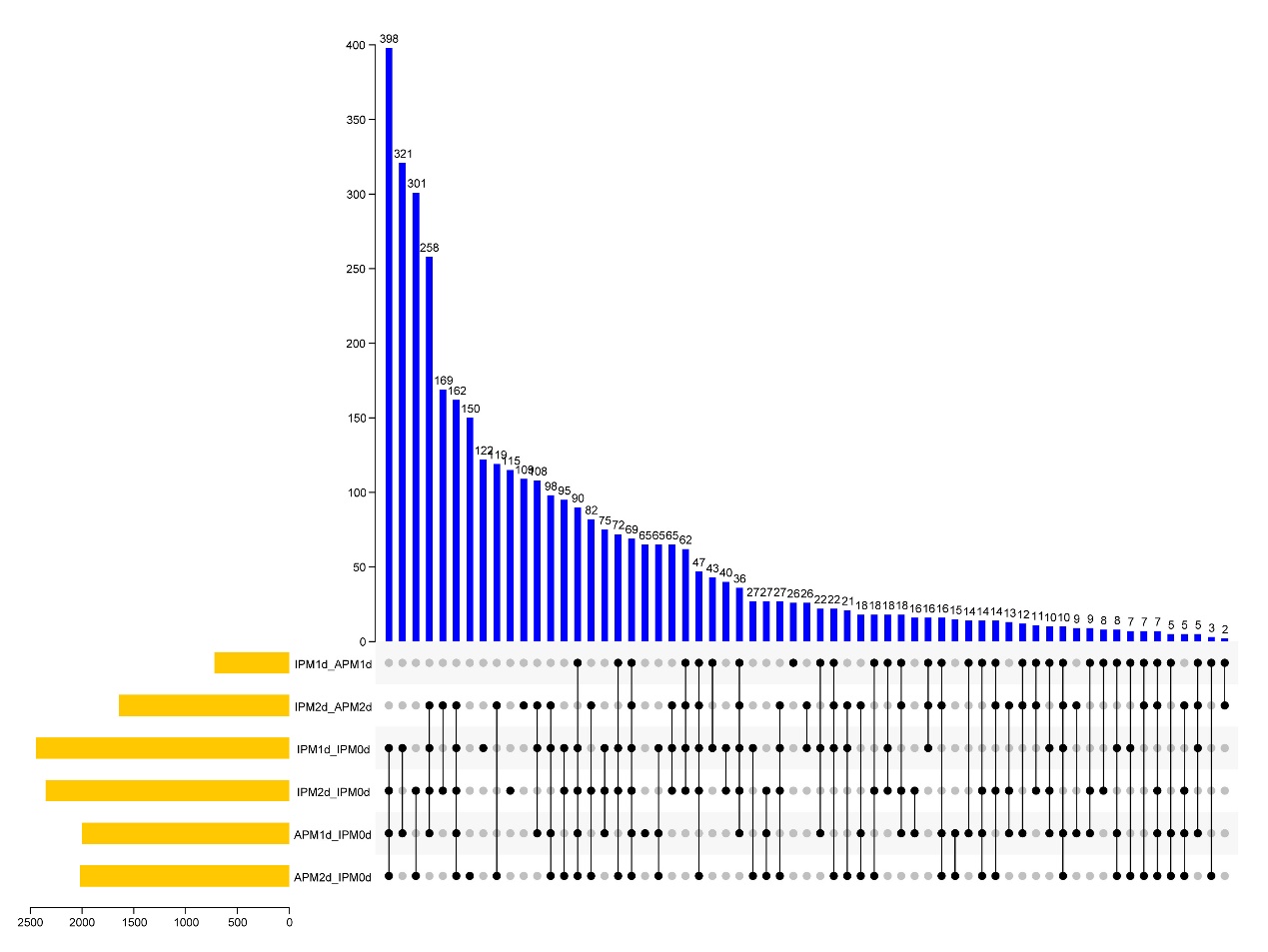


**Supplementary Figure 3.** Comparison of differentially expressed genes between transcriptome samples. The yellow horizontal histogram at the bottom left represents the number of differentially expressed genes between the two groups of samples. The black dot and line at the bottom right indicates which groups of differentially expressed genes correspond to the current blue histogram. The upper blue histogram represents the number of differentially expressed genes at the intersection.


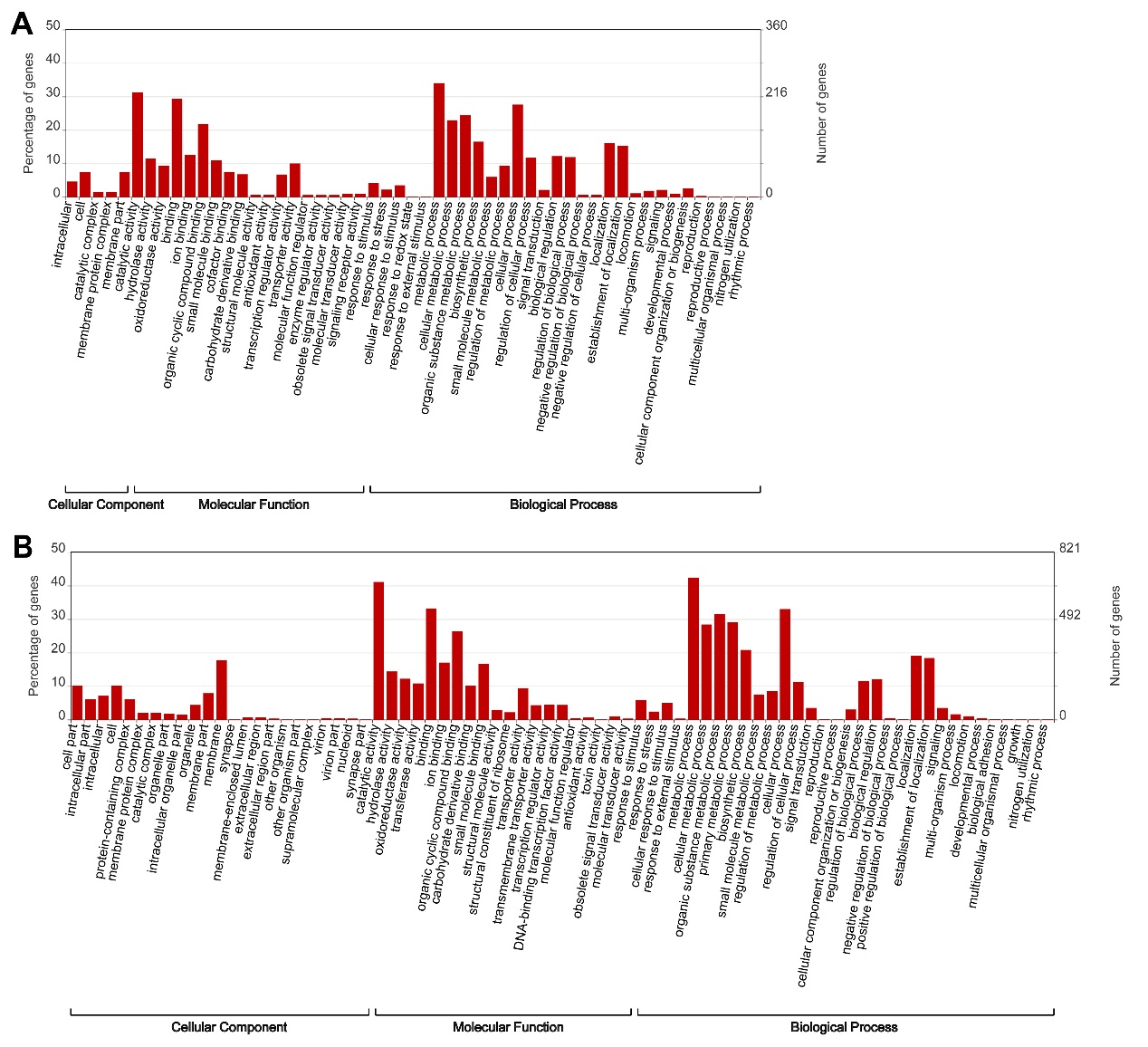


**Supplementary Figure 4.** GO enrichment analysis of differentially expressed genes between groups IPM and AMP. **(A)**, 1d; **(B)**, 2d. IPM, insoluble phosphorus medium; APM, available phosphorus medium.


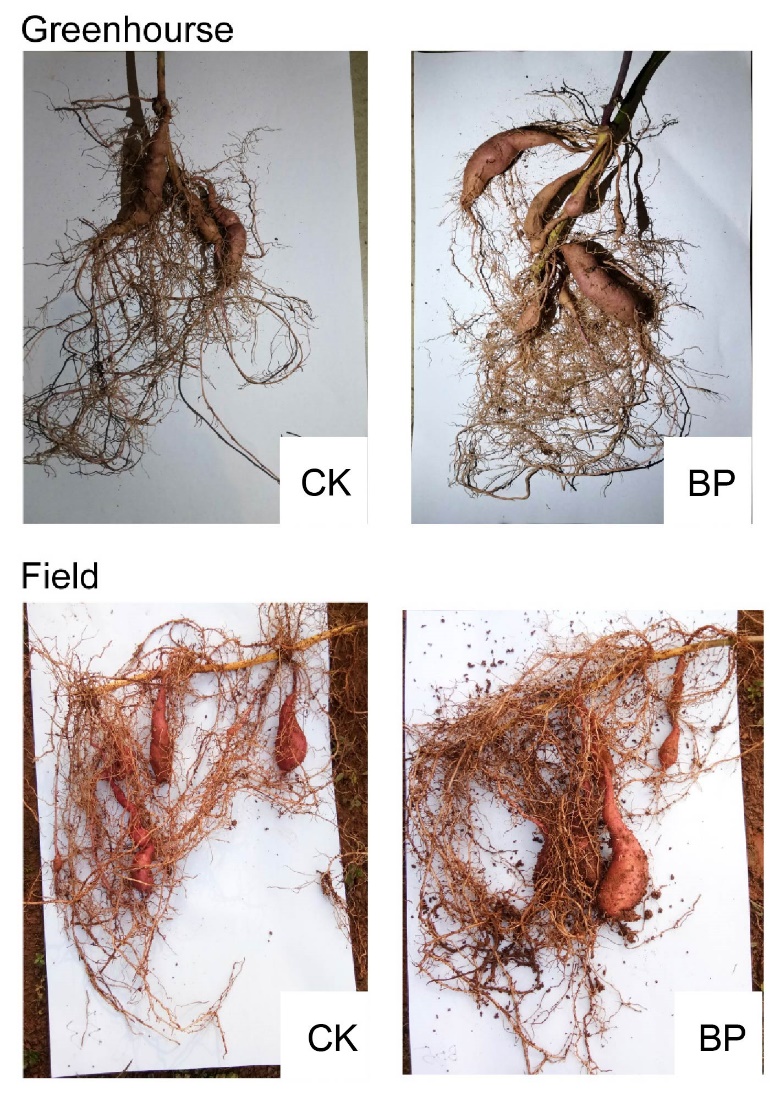


**Supplementary Figure 5.** Sweetpotato in greenhouse and field pot experiments. CK group, control group without phosphate-solubilizing bacteria 100 d; BP group, mixed phosphate-solubilizing bacteria was applied 100 d.

**Supplementary Tables**

**Supplementary Table 1.** The barren rocky soil properties

| Soil type | pH | TC (g/kg) | DOC (mg/kg) | TN (g/kg) | AN (mg/kg) | TP (g/kg) | AP (mg/kg) | TK (g/kg) | AK (g/kg) |
| --- | --- | --- | --- | --- | --- | --- | --- | --- | --- |
| purplish soils | 8.5 | 16.2 | 89.2 | 0.5 | 7.5 | 0.6 | 1.1 | 17.5 | 163.6 |

**Supplementary Table 2.** Diameter of dissolved phosphorus circle

|  | D1（mm） | D2（mm） | Average diameter（mm） | D/d |
| --- | --- | --- | --- | --- |
| BP1 | — | — | — | — |
| BP2 | — | — | — | — |
| BP3 | — | — | — | — |
| BP4 | — | — | — | — |
| BP5 | — | — | — | — |
| BP6 | — | — | — | — |
| BP10 | 22.00 | 23.00 | 22.50 | 3.75 |
| BP11 | 26.00 | 25.50 | 25.75 | 4.29 |
| BP17 | — | — | — | — |
| BP20 | 11.00 | 12.00 | 11.50 | 1.92 |
| BP21 | 7.00 | 7.000 | 7.00 | 1.17 |
| BP22 | 8.00 | 7.00 | 7.50 | 1.25 |
| BP23 | 25.00 | 24.50 | 24.75 | 4.13 |
| BP25 | 7.00 | 7.00 | 7.00 | 1.17 |
| BP29 | — | — | — | — |
| FP1 | 11.50 | 10.50 | 11.00 | 1.83 |
| FP2 | 15.00 | 15.00 | 15.00 | 2.50 |
| FP3 | 21.50 | 22.00 | 21.75 | 3.63 |
| FP4 | — | — | — | — |
| FP12 | 23.50 | 24.00 | 23.75 | 3.96 |
| FP16 | 10.00 | 11.20 | 10.60 | 1.77 |
| FP17 | 11.00 | 12.00 | 11.50 | 1.92 |
| FP18 | 8.00 | 9.00 | 8.50 | 1.42 |
| FP19 | 6.50 | 6.50 | 6.50 | 1.08 |
| FP20 | — | — | — | — |
| FP23 | 6.50 | 7.00 | 6.75 | 1.13 |

D, diameter of dissolved phosphorus circle; d, diameter of the round hole, 6.00 mm; D/d, Ratio of diameter of dissolved phosphorus circle to diameter of the round hole.

**Supplementary Table 3.** Phosphorus solubility of phosphorus-solubilizing bacteria

|  | Phosphorus-solubilizing ability (mg/L) | Tukey HSD test |
| --- | --- | --- |
| FP12 | 744.00 ± 4.00 | a |
| FP16 | 432.67 ± 5.51 | b |
| FP18 | 337.67 ± 29.77 | c |
| BP11 | 326.67 ± 22.74 | cd |
| BP23 | 309.33 ± 34.95 | cde |
| FP2 | 305.33 ± 2.31 | cde |
| FP19 | 298.00 ± 1.73 | cdef |
| BP10 | 274.67 ± 26.63 | defgh |
| FP17 | 264.33 ± 20.23 | efghi |
| BP20 | 245.33 ± 12.22 | fghij |
| BP6 | 230.33 ± 48.21 | ghij |
| FP20 | 226.67 ± 10.21 | hij |
| FP23 | 224.00 ± 8.54 | ij |
| FP3 | 206.67 ± 2.31 | j |
| BP1 | 151.67 ± 4.93 | k |
| BP22 | 151.33 ± 17.16 | k |
| FP1 | 150.67 ± 10.07 | k |
| BP2 | 147.67 ± 15.31 | kl |
| BP17 | 136.67 ± 15.89 | kl |
| BP3 | 123.33 ± 2.89 | kl |
| BP25 | 121.67 ± 4.51 | kl |
| BP4 | 117.67 ± 2.52 | klm |
| BP5 | 115.67 ± 7.57 | klm |
| FP4 | 102.67 ± 16.92 | klm |
| BP29 | 94.67 ± 7.51 | lm |
| BP21 | 65.00 ± 7.00 | mn |
| CK7d | 38.33 ± 5.13 | n |
| CK0d | 21.33 ± 4.04 | n |

CK0d, control 0 d; CK7d, control 7d. The values were average ± SD. Different letters indicated significant difference (*P* <0.05).

**Supplementary Table 4.** Identification of phosphorus-solubilizing bacteria using 16S rRNA gene

|  | Length (bp) | Coverage (%) | Identity (%) | E value | Species |
| --- | --- | --- | --- | --- | --- |
| BP10 | 1446 | 99 | 99.44 | 0 | *Stenotrophomonas maltophilia* |
| BP11 | 1431 | 98 | 98.94 | 0 | *Achromobacter xylosoxidans* |
| BP23 | 1416 | 99 | 99.08 | 0 | *Achromobacter pulmonis* |
| FP2 | 1448 | 99 | 99.44 | 0 | *Stenotrophomonas maltophilia* |
| FP12 | 1382 | 98 | 98.83 | 0 | *Ochrobactrum haematophilum* |
| FP16 | 1427 | 98 | 99.36 | 0 | *Cellulosimicrobium cellulans* |

**Supplementary Table 5.** Comparison of phosphorus-solubilizing bacteria FP12 and *Ochrobactrum* genomes

| Bacterial strains | Total lenght (bp) | GC (%) | ANI value (%) respect to FP12 | |
| --- | --- | --- | --- | --- |
| FP12 | 4916912 | 57.06 | | 100 |
| *O. haematophilum* strain FI11154 | 5482303 | 57.02 | | 97.84 |
| *O. haematophilum* ASM593810v1 | 5503262 | 56.67 | | 89.22 |
| *O. haematophilum* ASM355013v1 | 4910164 | 59.13 | | 85.42 |
| *Ochrobactrum lupini* strain LUP21 | 5582813 | 56.35 | | 80.97 |
| *Ochrobactrum* sp. 3-3 | 4951322 | 57.3 | | 80.94 |
| *Ochrobactrum anthropi* ATCC 49188 | 5205777 | 56.13 | | 80.84 |
| *Ochrobactrum intermedium* LMG 3301 | 4725392 | 57.74 | | 80.82 |
| *Ochrobactrum pecoris* strain 08RB2639 | 5057340 | 55.95 | | 80.8 |
| *Ochrobactrum anthropi* CTS-325 | 4726982 | 56.02 | | 80.74 |
| *Ochrobactrum oryzae* strain OA447 | 4467006 | 56.21 | | 80.74 |
| *Ochrobactrum cytisi* strain IPA7.2 | 5965364 | 55.37 | | 80.35 |
| *Ochrobactrum thiophenivorans* strain MYb6 | 4657555 | 53.41 | | 78.5 |
| *Ochrobactrum pituitosum* strain CCUG 50899 | 5518186 | 53.42 | | 78.47 |
| *Ochrobactrum grignonense* strain OgA9a | 4838271 | 54.15 | | 78.36 |
| *Ochrobactrum* sp. MYb14 | 4632136 | 53.42 | | 78.36 |
| *Ochrobactrum* sp. A44 | 5645291 | 53.16 | | 78.01 |
| *Ochrobactrum rhizosphaerae* strain PR17 | 4904011 | 53.01 | | 77.97 |

ANI, Average Nucleotide Identity.

**Supplementary Table 6.** The expression level of genes related to organic acids metabolism and acid tolerance

|  | gene id | IPM0d | IPM1d | IPM2d | APM1d | APM2d |
| --- | --- | --- | --- | --- | --- | --- |
| GDH | FP12_GM000535 | 1681.81±288.31 | 654.04±75.80 | 236.94±3.84 | 464.11±3.06 | 448.71±1.43 |
| PQQ | FP12_GM003320 | 161.83±6.32 | 170.58±2.28 | 685.11±42.45 | 161.62±8.21 | 606.09±6.89 |
|  | FP12_GM003321 | 136.39±9.15 | 136.68±8.95 | 333.55±18.39 | 131.81±2.32 | 180.45±19.18 |
|  | FP12_GM003322 | 16.34 ±3.03 | 11.40±3.68 | 100.94±18.89 | 15.97±0.95 | 78.18±9.28 |
|  | FP12_GM003323 | 66.77±5.97 | 59.55±2.34 | 155.55±4.81 | 57.37±6.63 | 132.66±18.54 |
| CS | FP12_GM001724 | 419.42±43.41 | 1436.69±164.64 | 423.74±37.03 | 1408.09±64.76 | 390.95±5.48 |
| ACO | FP12_GM000657 | 951.22±220.95 | 2161.97±185.71 | 7516.39±115.89 | 1842.70±49.09 | 4198.88±522.90 |
| IDH | FP12_GM001676 | 400.11±78.00 | 908.69±161.91 | 114.40±17.38 | 832.12±71.77 | 94.25±23.82 |
| OGDH | FP12_GM004383 | 458.02±42.23 | 899.76±109.93 | 1101.11±25.06 | 782.58±23.26 | 808.89±17.13 |
| SUC | FP12_GM004382 | 355.91±15.52 | 2215.90±275.87 | 1488.90±100.04 | 1754.19±216.18 | 1179.57±87.53 |
| SDH | FP12_GM004404 | 338.60±76.46 | 1173.84±34.80 | 780.96±36.27 | 979.35±76.41 | 432.90±12.51 |
| FH | FP12_GM002133 | 257.16±65.75 | 309.45±39.74 | 102.62±2.98 | 212.49±7.01 | 135.33±11.97 |
| MDH | FP12_GM004380 | 224.44±42.29 | 671.88±151.91 | 217.49±7.18 | 1088.42±164.56 | 309.99±27.07 |
| POX | FP12_GM000868 | 212.25±27.52 | 210.15±28.33 | 132.08±3.63 | 161.17±4.76 | 167.66±15.61 |
| LDH | FP12_GM004244 | 100.51±3.02 | 358.87±65.47 | 64.54±4.41 | 332.75±34.03 | 60.60±2.44 |
| RpoS | FP12_GM001399 | 1521.73±136.14 | 2680.65±414.61 | 5540.95±93.00 | 1371.87±113.45 | 3997.41±627.88 |
| PhoP | FP12_GM002495 | 52.18±6.66 | 127.72±25.60 | 110.48±48.17 | 133.34±25.40 | 74.46±3.08 |
| Fur | FP12_GM000775 | 1702.51±208.17 | 2637.18±23.51 | 583.90±30.36 | 1425.55±97.32 | 477.64±87.90 |
| OmpR | FP12_GM004046 | 518.86±61.11 | 584.40±48.67 | 414.22±34.21 | 556.62±50.89 | 223.08±24.45 |

The values represent average±SD.

**Supplementary Table 7.** The description of experiment groups

| Groups | Description | Experiment |
| --- | --- | --- |
| CK0 | control group 0 d | Treating the barren rocky soil with PSB |
| CK10 | control group 10 d | Treating the barren rocky soil with PSB |
| MB | mixed phosphate-solubilizing bacteria | Treating the barren rocky soil with PSB |
| CK0 | control group 0 d | Addition of sweetpotato root exudates |
| BCK | control group without mixed phosphate-solubilizing bacteria 10 d | Addition of sweetpotato root exudates |
| BW | mixed phosphate-solubilizing bacteria with sterile water | Addition of sweetpotato root exudates |
| BR | mixed phosphate-solubilizing bacteria with sweetpotato root exudates | Addition of sweetpotato root exudates |
| CK0 | control group 0 d | Pot experiments |
| CK | control group without phosphate-solubilizing bacteria 100 d | Pot experiments |
| BP | mixed phosphate-solubilizing bacteria was applied 100 d | Pot experiments |
